# Supplementary figures and images for: The Novel Association of Early Apoptotic Circulating Tumor Cells with Treatment Outcomes in Breast Cancer Patients
Source: Int J Mol Sci. 2022 Aug 22;23(16):9475. doi: 10.3390/ijms23169475 (PMC9408919; doi:10.3390/ijms23169475)

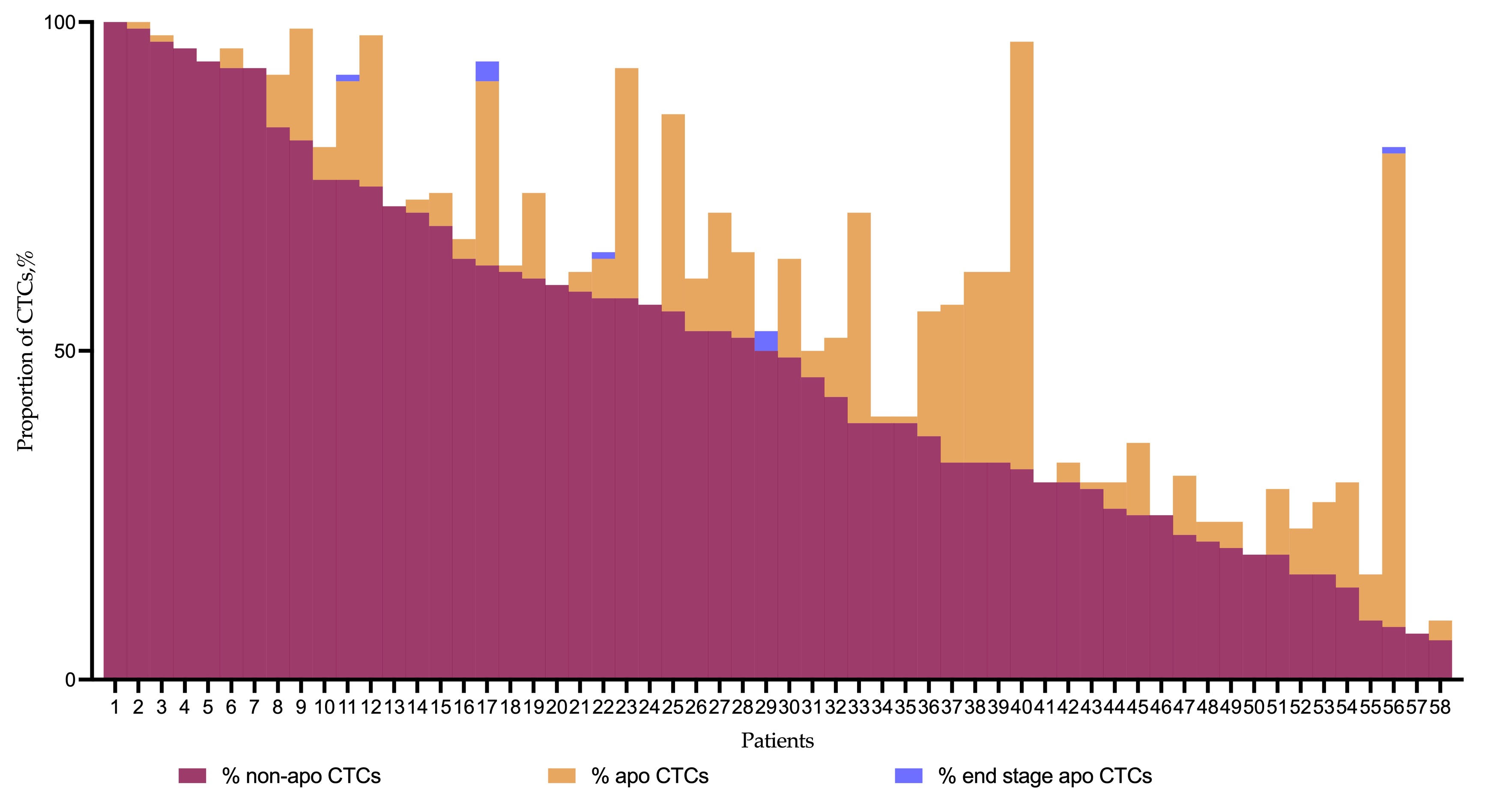

Supplement: Supplementary file 1 [file ijms-23-09475-s001.zip › ijms-1854919-supplementary.jpeg]
